# Use of Colorectal Cancer Screening Tests by State

**DOI:** 10.5888/pcd15.170535

**Published:** 2018-06-14

**Authors:** Djenaba A. Joseph, Jessica B. King, Thomas B. Richards, Cheryll C. Thomas, Lisa C. Richardson

**Affiliations:** 1 Division of Cancer Prevention and Control, Centers for Disease Control and Prevention, Atlanta, Georgia

**Figure:**
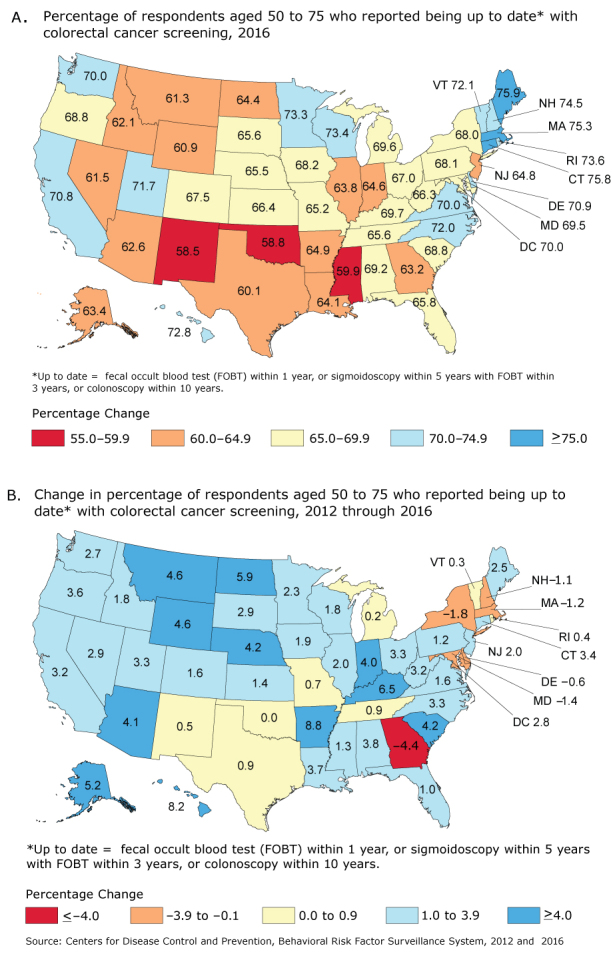
Progress toward increased use of colorectal cancer (CRC) screening tests, by state. A. Percentage of respondents aged 50 to 75 who reported being up to date with CRC screening in the 2016 Behavioral Risk Factor Surveillance System (1). The percentage up to date for the United States overall was 67.3%. B. The absolute change in percentage of respondents aged 50 to 75 who reported being up to date with CRC screening from 2012 through 2016, by state, Behavioral Risk Factor Surveillance System, 2012 (2), 2016 (1). Up to date is defined as having had a fecal occult blood test (FOBT) within the past year, sigmoidoscopy within the past 5 years with FOBT within the past 3 years, or colonoscopy within the past 10 years. Source: CDC Behavioral Risk Factor Surveillance System (BRFSS), BRFSS, 2012 and 2016 (1–2). **Table:** 

State	Percentage of Respondents Aged 50–75 y Up to Date With CRC Screening, 2016	Absolute Change, Percentage of Respondents Aged 50–75 y Up to Date With CRC Screening, 2012 through 2016
Alabama	69.2	3.8
Alaska	63.4	5.2
Arizona	62.6	4.1
Arkansas	64.9	8.8
California	70.8	3.2
Colorado	67.5	1.6
Connecticut	75.8	3.4
Delaware	70.9	−0.6
Florida	65.8	1.0
Georgia	63.2	−4.4
Hawaii	72.8	8.2
Idaho	62.1	1.8
Illinois	63.8	2.0
Indiana	64.6	4.0
Iowa	68.2	1.9
Kansas	66.4	1.4
Kentucky	69.7	6.5
Louisiana	64.1	3.7
Maine	75.9	2.5
Maryland	69.5	–1.4
Massachusetts	75.3	−1.2
Michigan	69.6	0.2
Minnesota	73.3	2.3
Mississippi	59.9	1.3
Missouri	65.2	0.7
Montana	61.3	4.6
Nebraska	65.5	4.2
Nevada	61.5	2.9
New Hampshire	74.5	–1.1
New Jersey	64.8	2.0
New Mexico	58.5	0.5
New York	68.0	–1.8
North Carolina	72.0	3.3
North Dakota	64.4	5.9
Ohio	67.0	3.3
Oklahoma	58.8	0
Oregon	68.8	3.6
Pennsylvania	68.1	1.2
Rhode Island	73.6	0.4
South Carolina	68.8	4.2
South Dakota	65.6	2.9
Tennessee	65.6	0.9
Texas	60.1	0.9
Utah	71.7	3.3
Vermont	72.1	0.3
Virginia	70.0	1.6
Washington	70.0	2.7
West Virginia	66.3	3.2
Wisconsin	73.4	1.8
Wyoming	60.9	4.6
District of Columbia	70.0	2.8

## Background

Colorectal cancer (CRC) is the second most common cause of cancer death among cancers that affect both men and women (3). There is strong evidence that screening reduces CRC incidence and deaths from the disease (4). The 2008 US Preventive Services Task Force (USPSTF) recommendations include several test options for screening for CRC among adults aged 50 to 75: 1) annual high-sensitivity fecal occult blood test (FOBT), 2) colonoscopy every 10 years, or 3) sigmoidoscopy every 5 years with FOBT every 3 years (4). Despite strong evidence for its use, an estimated 23 million age-eligible adults were not tested for CRC in 2012 (5). This article describes the estimated percentage of adults aged 50 to 75 (eligible adults) who reported being up to date with CRC screening in 2016, and the change in the percentage from 2012 through 2016, by state.

## Methods

The Behavioral Risk Factor Surveillance Survey (BRFSS) is an annual, state-based, random-digit–dialed telephone survey of the civilian, noninstitutionalized adult population aged 18 or older. BRFSS collects information on health risk, behaviors, preventive-health practices, and health care access in the United States. Respondents aged 50 or older respond to questions asking if they ever had a CRC screening test and, if yes, when the most recent test was done. We analyzed data from the 2012 and 2016 BRFSS to estimate the percentage of adults aged 50 to 75 who reported having been screened for CRC screening consistent with 2008 USPSTF recommendations (up to date with CRC screening) by state (5). USPSTF recommendations were updated in 2016, but we used 2008 recommendations for our analysis. Respondents who declined to answer, had a missing answer, or who answered, “don’t know/not sure,” were excluded from the analysis. SUDAAN (RTI International) was used to account for the complex sampling design. Data were weighted to the age, sex, and racial/ethnic distribution of each state’s adult population by using intercensal estimates that were age-standardized to the 2016 BRFSS population. ArcGIS Desktop, version 10.5 (ESRI) was used to create a map series to show the percentage up to date in 2016 (Map A) and the absolute change in the percentage up to date between 2012 and 2016 (Map B).

## Main Findings

The percentage of adults aged 50 to 75 who reported being up to date with CRC screening in the United States increased from 65.5% in 2012 to 67.3% in 2016. The percentage of eligible adults who were up to date with CRC screening by state ranged from 58.5% (New Mexico) to 75.9% (Maine) in 2016 (Map A).

From 2012 through 2016, 37 states had an estimated increase of 1% or more in the percentage of eligible adults who were up to date with CRC screening (Map B), with the largest in Arkansas (8.8%), followed by Hawaii (8.2%), and 10 states ranging from 4% to 6.5%. Six states had an overall estimated decrease in the percentage that were up to date with the largest decline in Georgia (−4.4%). Of the 10 states with at least 70% of eligible adults up to date in 2012, 4 had an estimated increase of 1% or more, and 4 had an overall estimated decline by 2016. Of the 15 states with 65% to 69.9% of eligible adults up to date in 2012, 12 had an estimated increase of 1% or more, and 2 had an overall estimated decline. Of the 15 states with 60% to 64.9% of eligible adults up to date in 2012, 13 had an estimated increase of 1% or more, and 4 had an estimated increase of 4% or more. Finally, of 11 states with less than 60% of eligible adults up to date in 2012, 8 had an estimated increase of 1% or more, and 6 had an estimated increase of 4% or more.

## Action

Most states had an estimated increase in the percentage of eligible adults who were up to date with CRC screening from 2012 to 2016 for an overall estimated increase in CRC screening prevalence of 1.8%; this represents an estimated additional 5,095,254 people who were screened. The largest estimated gains were in states with lower (<65%) percentages of eligible adults who were up to date in 2012, whereas smaller estimated increases or declines occurred in states with higher (≥70%) percentages of eligible adults who were up to date. This suggests that as the percentage of people up to date with CRC screening increases in states, the remaining unscreened, eligible adults may be harder to reach. Previous research described several barriers to CRC screening, including lack of health insurance or a regular health care provider, failure of the provider to recommend screening, the patient’s lack of awareness or knowledge of the need to be screened, low household income, low educational attainment, and being of a racial/ethnic minority (6). Public health can play a critical role in linking hard-to-reach populations to health care systems. CDC’s Colorectal Cancer Control Program (https://www.cdc.gov/cancer/crccp/) funds 23 states, 6 universities, and a tribe to partner with primary care clinics that serve hard-to-reach populations to support the implementation of evidence-based interventions recommended by the Community Guide (https://www.thecommunityguide.org/topic/cancer) that have been shown to increase CRC screening. These programs leverage public health expertise in population health management by working with clinics to embed sustainable cancer screening processes.

Our study has 4 limitations. First, CRC screening prevalence might be overestimated or underestimated, because BRFSS does not specify whether testing was done for screening or diagnosis. Second, data were self-reported and may be subject to recall and social desirability bias. Third, our analysis did not account for sampling error, and described changes may be due to sampling error alone. Fourth, response rates were low (45.2% in 2012 and 47.1% in 2016), and respondents who did not answer all questions were excluded (6.8% in 2012, 8.9% in 2016), although BRFSS weighting procedure attempts to correct for nonresponse.

Fourteen states in the United States have reached the Healthy People 2020 objective for being up to date with CRC screening (70.5%), and most remaining states have made progress toward reaching this goal. Continued support for state and local efforts to increase CRC screening, such CDC’s Comprehensive Cancer Control Coalitions, is needed. Efforts to integrate these interventions into other preventive care and disease management practices may improve their reach and sustainability.
